# COVID-19 Vaccine Second-Dose Completion and Interval Between First and Second Doses Among Vaccinated Persons — United States, December 14, 2020−February 14, 2021

**DOI:** 10.15585/mmwr.mm7011e2

**Published:** 2021-03-19

**Authors:** Jennifer L. Kriss, Laura E. Reynolds, Alice Wang, Shannon Stokley, Matthew M. Cole, LaTreace Q. Harris, Lauren K. Shaw, Carla L. Black, James A. Singleton, David L. Fitter, Dale A. Rose, Matthew D. Ritchey, Robin L. Toblin, CDC COVID-19 Vaccine Task Force

**Affiliations:** 1 CDC COVID-19 Response Team.

In December 2020, two COVID-19 vaccines (Pfizer-BioNTech and Moderna) received Emergency Use Authorization from the Food and Drug Administration.*^,^^†^ Both vaccines require 2 doses for a completed series. The recommended interval between doses is 21 days for Pfizer-BioNTech and 28 days for Moderna; however, up to 42 days between doses is permissible when a delay is unavoidable.^§^ Two analyses of COVID-19 vaccine administration data were conducted among persons who initiated the vaccination series during December 14, 2020−February 14, 2021, and whose doses were reported to CDC through February 20, 2021. The first analysis was conducted to determine whether persons who received a first dose and had sufficient time to receive the second dose (i.e., as of February 14, 2021, >25 days from receipt of Pfizer-BioNTech vaccine or >32 days from receipt of Moderna vaccine had elapsed) had received the second dose. A second analysis was conducted among persons who received a second COVID-19 dose by February 14, 2021, to determine whether the dose was received during the recommended dosing interval, which in this study was defined as 17–25 days (Pfizer-BioNTech) and 24–32 days (Moderna) after the first dose. Analyses were stratified by jurisdiction and by demographic characteristics. In the first analysis, among 12,496,258 persons who received the first vaccine dose and for whom sufficient time had elapsed to receive the second dose, 88.0% had completed the series, 8.6% had not received the second dose but remained within the allowable interval (≤42 days since the first dose), and 3.4% had missed the second dose (outside the allowable interval, >42 days since the first dose). The percentage of persons who missed the second dose varied by jurisdiction (range = 0.0%−9.1%) and among demographic groups was highest among non-Hispanic American Indian/Alaska Native (AI/AN) persons (5.1%) and persons aged 16−44 years (4.0%). In the second analysis, among 14,205,768 persons who received a second dose, 95.6% received the dose within the recommended interval, although percentages varied by jurisdiction (range = 79.0%−99.9%). Public health officials should identify and address possible barriers to completing the COVID-19 vaccination series to ensure equitable coverage across communities and maximum health benefits for recipients. Strategies to ensure series completion could include scheduling second-dose appointments at the first-dose administration and sending reminders for second-dose visits.

During December 14, 2020−February 14, 2021, a total of 40,517,900 persons initiated the COVID-19 vaccination series and had vaccine administration data reported to CDC by February 20, 2021. Providers submitted COVID-19 vaccine administration data to CDC via immunization information systems (IIS), the Vaccine Administration Management System (VAMS), or direct data submission.^¶^ First and second doses were linked based on a recipient ID assigned by the reporting entity (e.g., jurisdictions, territories, and federal entities) and the three-digit reporting entity code.**

Two analyses were conducted that included 58 jurisdictions (49 states, the District of Columbia, and eight territories or freely associated states^††^; 37,421,619 persons) (Figure). The first analysis assessed the series completion status among 12,496,258 persons who had received the first vaccine dose and for whom sufficient time had elapsed to receive the second dose. The second analysis examined the interval between the first and second doses among 14,205,768 persons who had received a second dose. Persons for whom the manufacturer of the first dose was unknown (0.2%; 86,480) were excluded from both analyses. Persons with mismatched vaccine manufacturers for the first and second doses (0.2%; 90,484) were categorized according to the first-dose manufacturer’s recommended vaccination schedule. Persons for whom sufficient time to receive the second dose had not elapsed were excluded from analysis of completion status (66.4%; 24,838,881); persons who did not receive a second dose by February 14, 2021, were excluded from the second-dose interval analysis (61.8%; 23,129,371).

**FIGURE:**
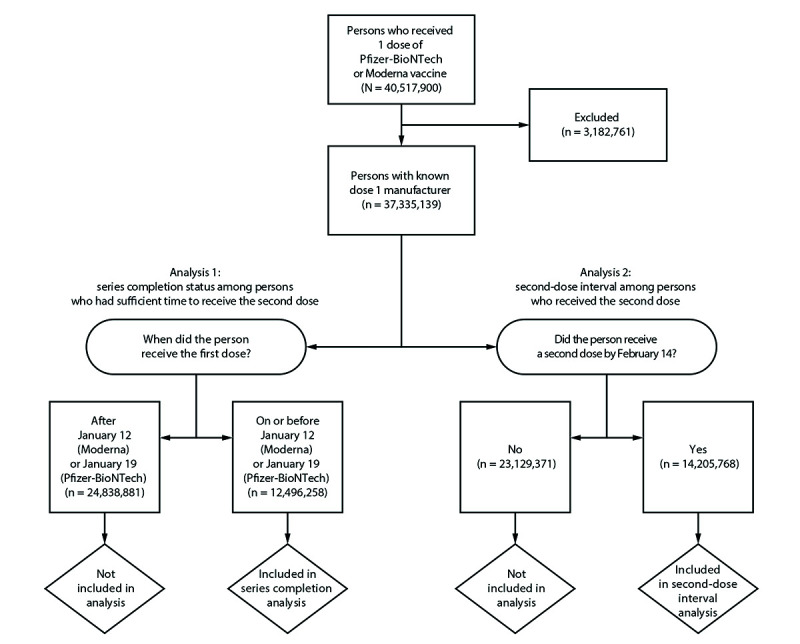
Inclusion criteria for analysis of COVID-19 vaccine series completion and second-dose interval* — United States,† December 14, 2020–February 14, 2021 * The recommended interval between the first and second dose is 21 days for Pfizer-BioNTech and 28 days for Moderna; in this study, second doses received 17–25 days (Pfizer-BioNTech) and 24–32 days (Moderna) after the first dose were included. ^†^ Texas did not submit individual-dose vaccination data; therefore, persons who received ≥1 dose in Texas (n = 3,096,281) were excluded; persons for whom the manufacturer of the first dose was unknown (from any jurisdiction) were also excluded (n = 86,480).

To assess second-dose completion status, persons who had sufficient time to receive the second dose (i.e., received the first dose on or before January 12 [Moderna] or January 19 [Pfizer-BioNTech] and >32 days or >25 days, respectively, had elapsed between the first dose and February 14) were included and categorized into three mutually exclusive groups: 1) completed series (received 2 doses on separate days within any time interval); 2) no second dose received but remained within the allowable interval (26–42 days [Pfizer-BioNTech] or 33–42 days [Moderna] after first dose); or 3) missed the second dose (>42 days after first dose) (Supplementary Figure 1, https://stacks.cdc.gov/view/cdc/103854).

To examine the interval between doses, persons who received the second dose at any time during December 14, 2020−February 14, 2021, were categorized into four mutually exclusive groups according to timing of receipt of the second dose: 1) early (before the recommended interval), 2) during the recommended interval, 3) after the recommended interval but within the allowable interval, or 4) late (outside the allowable interval). Both analyses were conducted at the national level and analyzed by jurisdiction and demographic characteristics (race/ethnicity, age, and sex) using information reported with the first-dose record. Persons with missing data for race/ethnicity, age, or sex were excluded from the respective demographic analyses. Analyses were conducted using SAS (version 9.4; SAS Institute). This activity was reviewed by CDC and was conducted consistent with applicable federal law and CDC policy.^§§^

Among 12,496,258 persons who received a first COVID-19 vaccine dose and for whom sufficient time to receive the second dose had elapsed (Figure), 88.0% had completed the series, 8.6% had not received the second dose but remained within the allowable interval, and 3.4% had missed the second dose (Table 1). Substantial differences in completion status were observed across jurisdictions (median = 88.9%, range = 75.3%−99.7%) (Table 2) (Supplementary Figure 2, https://stacks.cdc.gov/view/cdc/103854). In 10 jurisdictions, <85%^¶¶^ of persons who received a first dose had completed the series. In addition, the percentage of persons who missed the second dose varied by jurisdiction, ranging from 0.0% to 9.1% (median = 2.8%), with >5% of persons having missed the second dose in eight jurisdictions.

**TABLE 1:** Second-dose completion status and interval between first and second dose among persons who initiated the COVID-19 vaccination series, by vaccine manufacturer — United States, December 14, 2020–February 14, 2021*

Completion status and dosing interval	No. (%)^†^		
	Total	Pfizer-BioNTech	Moderna
**Received ≥1 dose**	**37,335,139**	**18,161,871**	**19,173,268**
**Completion status^§^ among persons with sufficient time to receive second dose**	**12,496,258**	7,750,089	4,746,169
Completed series	**10,999,097 (88.0)**	6,791,301 (87.6)	4,207,796 (88.7)
No second dose but remained within allowable interval^¶^	**1,078,336 (8.6)**	693,650 (9.0)	384,686 (8.1)
Missed second dose**	**418,825 (3.4)**	265,138 (3.4)	153,687 (3.2)
**Dosing interval among persons who received second dose**	**14,205,768**	8,400,210	5,805,558
Early**^††^**	**216,905 (1.5)**	98,585 (1.2)	118,320 (2.0)
During recommended interval^§§^	**13,582,544 (95.6)**	8,053,661 (95.9)	5,528,883 (95.2)
After recommended interval but within allowable interval^¶¶^	**392,935 (2.8)**	240,329 (2.9)	152,606 (2.6)
Late***	**13,384 (0.1)**	7,635 (0.1)	5,749 (0.1)

* Vaccines administered during December 14, 2020–February 14, 2021, and reported to CDC by February 20, 2021.

^†^ Because of rounding, column percentages might not sum to 100%.

^§^ Among persons who received their first dose on or before January 19 for Pfizer-BioNTech (>25 days between the first dose and February 14) or January 12 for Moderna (>32 days between the first dose and February 14).

^¶^ 26–42 days (Pfizer-BioNTech) or 33–42 days (Moderna) after first dose; no second dose received.

** >42 days after first dose; no second dose received.

^††^ Received second dose <17 days (Pfizer-BioNTech) or <24 days (Moderna) after first dose.

^§§^ Received second dose 17–25 days (Pfizer-BioNTech) or 24–32 days (Moderna) after first dose.

^¶¶^ Received second dose 26–42 days (Pfizer-BioNTech) or 33–42 days (Moderna) after first dose.

*** Received second dose >42 days after first dose.

**TABLE 2:** Second-dose completion status and interval between first and second dose among persons who initiated the COVID-19 vaccination series, by jurisdiction and demographic characteristics — United States, December 14, 2020–February 14, 2021*

Jurisdiction and demographic characteristic	Completion status^†^ among persons with sufficient time to receive second dose (%)				Dosing interval among persons who received second dose (%)				
	No.	Completed series	No second dose but remained within allowable interval^§^	Missed second dose^¶^	No.	Early**	During recommended interval^††^	After recommended interval but within allowable interval^§§^	Late^¶¶^
**Total, no. (%)**	**12,496,258**	**10,999,097 (88.0)**	**1,078,336 (8.6)**	**418,825 (3.4)**	**14,205,768**	**216,905 (1.5)**	**13,582,544 (95.6)**	**392,935 (2.8)**	**13,384 (0.1)**
**State/Area**									
Alabama	**129,629**	(87.3)	(9.9)	(2.8)	**150,202**	(2.1)	(95.7)	(2.1)	(0.1)
Alaska	**57,293**	(90.5)	(6.9)	(2.6)	**68,344**	(0.9)	(97.9)	(1.2)	(0.0)
Arizona	**274,548**	(85.4)	(11.6)	(2.9)	**300,844**	(3.2)	(90.8)	(5.9)	(0.1)
Arkansas	**147,459**	(82.8)	(12.4)	(4.7)	**148,477**	(1.4)	(96.6)	(2.0)	(0.1)
California	**1,269,213**	(87.2)	(9.0)	(3.8)	**1,538,150**	(1.2)	(96.4)	(2.3)	(0.1)
Colorado	**280,523**	(94.2)	(4.1)	(1.7)	**328,338**	(0.9)	(97.4)	(1.6)	(0.1)
Connecticut	**234,761**	(82.9)	(14.5)	(2.6)	**256,784**	(7.5)	(86.4)	(5.3)	(0.8)
Delaware	**35,454**	(87.6)	(9.9)	(2.5)	**37,387**	(1.0)	(92.1)	(6.8)	(0.1)
District of Columbia	**45,206**	(85.6)	(10.4)	(4.0)	**46,137**	(1.3)	(96.4)	(2.2)	(0.0)
Florida	**1,094,586**	(87.8)	(9.1)	(3.1)	**1,218,397**	(2.0)	(95.4)	(2.6)	(0.1)
Georgia	**362,414**	(88.9)	(8.4)	(2.7)	**443,669**	(1.8)	(94.9)	(3.2)	(0.1)
Hawaii	**19,023**	(88.9)	(6.8)	(4.3)	**20,147**	(0.8)	(93.3)	(5.8)	(0.1)
Idaho	**54,880**	(93.1)	(4.9)	(2.0)	**70,856**	(1.4)	(97.2)	(1.3)	(0.1)
Illinois	**419,419**	(85.5)	(9.4)	(5.0)	**449,475**	(1.1)	(97.2)	(1.7)	(0.1)
Indiana	**275,381**	(90.3)	(8.7)	(1.0)	**312,681**	(1.0)	(96.8)	(2.1)	(0.1)
Iowa	**138,418**	(81.8)	(16.2)	(2.0)	**129,403**	(0.5)	(93.5)	(5.8)	(0.2)
Kansas	**116,535**	(87.0)	(7.6)	(5.4)	**122,686**	(2.0)	(94.6)	(3.4)	(0.1)
Kentucky	**199,398**	(84.0)	(10.2)	(5.8)	**210,892**	(0.6)	(96.1)	(3.3)	(0.1)
Louisiana	**234,624**	(94.4)	(4.7)	(0.9)	**274,481**	(0.9)	(98.3)	(0.8)	(0.0)
Maine	**62,633**	(89.2)	(5.8)	(4.9)	**68,479**	(0.3)	(98.2)	(1.3)	(0.1)
Maryland	**231,172**	(91.4)	(6.9)	(1.7)	**266,356**	(0.8)	(94.2)	(4.9)	(0.1)
Massachusetts	**294,106**	(82.1)	(11.4)	(6.6)	**305,083**	(1.3)	(96.6)	(2.0)	(0.1)
Michigan	**447,555**	(95.4)	(3.4)	(1.2)	**538,614**	(0.6)	(97.3)	(2.1)	(0.1)
Minnesota	**217,567**	(90.5)	(6.3)	(3.2)	**255,434**	(2.1)	(95.4)	(2.5)	(0.0)
Mississippi	**103,965**	(89.8)	(8.8)	(1.3)	**128,671**	(1.1)	(95.9)	(3.0)	(0.1)
Missouri	**216,227**	(93.0)	(4.5)	(2.5)	**265,192**	(0.6)	(96.9)	(2.4)	(0.1)
Montana	**50,572**	(90.9)	(5.4)	(3.6)	**57,032**	(1.4)	(95.4)	(3.1)	(0.2)
Nebraska	**94,464**	(90.3)	(5.4)	(4.3)	**102,101**	(0.7)	(96.5)	(2.7)	(0.1)
Nevada	**95,396**	(91.7)	(5.7)	(2.6)	**125,342**	(1.0)	(96.7)	(2.2)	(0.1)
New Hampshire	**84,323**	(93.5)	(4.2)	(2.3)	**95,776**	(8.7)	(82.3)	(8.3)	(0.7)
New Jersey	**324,161**	(87.7)	(9.1)	(3.1)	**388,744**	(0.6)	(97.5)	(1.9)	(0.0)
New Mexico	**142,008**	(87.4)	(8.5)	(4.1)	**163,459**	(1.4)	(96.0)	(2.5)	(0.1)
New York	**823,922**	(87.2)	(8.6)	(4.2)	**968,811**	(1.0)	(96.9)	(2.1)	(0.1)
North Carolina	**419,220**	(91.0)	(5.7)	(3.3)	**534,863**	(0.7)	(96.4)	(2.8)	(0.1)
North Dakota	**47,682**	(93.3)	(4.2)	(2.5)	**53,008**	(3.1)	(94.1)	(2.7)	(0.1)
Ohio	**436,574**	(89.6)	(7.7)	(2.7)	**515,733**	(1.4)	(95.0)	(3.5)	(0.1)
Oklahoma	**231,600**	(87.7)	(9.6)	(2.7)	**240,072**	(0.6)	(96.8)	(2.6)	(0.1)
Oregon	**189,028**	(92.9)	(5.5)	(1.6)	**213,812**	(0.6)	(96.7)	(2.6)	(0.1)
Pennsylvania	**479,037**	(87.6)	(9.2)	(3.2)	**522,255**	(1.6)	(96.0)	(2.4)	(0.1)
Rhode Island	**47,308**	(93.8)	(4.3)	(2.0)	**53,769**	(0.8)	(95.9)	(3.2)	(0.1)
South Carolina	**181,871**	(80.6)	(16.9)	(2.5)	**197,410**	(3.2)	(94.5)	(2.3)	(0.0)
South Dakota	**58,551**	(89.7)	(6.7)	(3.5)	**62,266**	(0.3)	(96.6)	(3.0)	(0.1)
Tennessee	**311,579**	(91.9)	(4.9)	(3.2)	**329,446**	(1.6)	(95.5)	(2.8)	(0.1)
Utah	**147,885**	(75.3)	(20.7)	(4.0)	**126,507**	(2.1)	(90.5)	(7.2)	(0.1)
Vermont	**36,563**	(91.9)	(5.3)	(2.8)	**38,759**	(1.2)	(97.6)	(1.2)	(0.0)
Virginia	**345,645**	(82.6)	(9.6)	(7.8)	**386,384**	(0.8)	(96.3)	(2.8)	(0.1)
Washington	**285,288**	(87.5)	(9.4)	(3.2)	**321,080**	(1.7)	(95.4)	(2.8)	(0.1)
West Virginia	**167,867**	(95.8)	(2.5)	(1.7)	**201,504**	(6.6)	(85.5)	(7.4)	(0.4)
Wisconsin	**216,685**	(93.5)	(5.2)	(1.3)	**260,163**	(0.6)	(96.5)	(2.9)	(0.0)
Wyoming	**27,746**	(88.2)	(9.5)	(2.3)	**31,486**	(1.4)	(95.8)	(2.8)	(0.1)
**Territories and freely associated states**									
American Samoa	**3,509**	(95.9)	(2.9)	(1.3)	**6,047**	(0.2)	(99.4)	(0.4)	(0.0)
Federated States of Micronesia	**577**	(85.1)	(14.9)	(0.0)	**895**	(1.0)	(79.0)	(20.0)	(0.0)
Guam	**10,447**	(87.0)	(8.3)	(4.7)	**12,557**	(0.5)	(98.9)	(0.6)	(0.0)
Marshall Islands	**707**	(76.8)	(14.1)	(9.1)	**725**	(0.4)	(98.3)	(1.2)	(0.0)
Northern Mariana Islands	**4,408**	(98.7)	(0.6)	(0.7)	**5,266**	(0.1)	(99.7)	(0.2)	(0.1)
Palau	**726**	(99.7)	(0.3)	(0.0)	**1,566**	(0.1)	(99.9)	(0.1)	(0.0)
Puerto Rico	**138,612**	(79.0)	(15.1)	(5.9)	**128,887**	(1.0)	(98.5)	(0.5)	(0.0)
U.S. Virgin Islands	**4,107**	(85.1)	(9.2)	(5.6)	**4,211**	(1.9)	(94.8)	(3.1)	(0.1)
**Demographic characteristics**									
Race/Ethnicity***	**6,764,604**	—	—	—	**8,002,280**	—	—	—	—
AI/AN, non-Hispanic	**145,449**	(83.7)	(11.2)	(5.1)	**148,823**	(1.5)	(96.0)	(2.4)	(0.1)
Asian, non-Hispanic	**365,379**	(90.2)	(7.0)	(2.8)	**428,595**	(1.2)	(95.9)	(2.8)	(0.1)
Black, non-Hispanic	**366,442**	(88.8)	(8.6)	(2.6)	**436,647**	(1.8)	(95.1)	(3.1)	(0.1)
Hispanic	**718,384**	(87.0)	(9.5)	(3.5)	**812,235**	(1.4)	(95.8)	(2.8)	(0.1)
Multiple/Other, non-Hispanic	**1,013,031**	(86.1)	(10.7)	(3.2)	**1,152,231**	(1.4)	(96.5)	(2.0)	(0.1)
NHPI, non-Hispanic	**17,755**	(90.3)	(6.8)	(2.9)	**20,042**	(1.0)	(96.1)	(2.7)	(0.1)
White, non-Hispanic	**4,138,164**	(90.3)	(7.4)	(2.3)	**5,003,707**	(1.7)	(95.0)	(3.1)	(0.1)
Age group, yrs	**12,489,174**	—	—	—	**14,202,212**	—	—	—	—
16–44	**4,507,276**	(88.1)	(7.9)	(4.0)	**4,653,564**	(1.3)	(95.0)	(3.6)	(0.1)
45–64	**4,211,791**	(88.7)	(7.7)	(3.6)	**4,453,503**	(1.4)	(95.5)	(3.0)	(0.1)
≥65	**3,770,107**	(87.2)	(10.5)	(2.3)	**5,095,145**	(1.9)	(96.3)	(1.8)	(0.0)
Sex	**12,128,929**	—	—	—	**13,866,864**	—	—	—	—
Female	**7,675,229**	(88.4)	(8.4)	(3.2)	**8,630,313**	(1.5)	(95.6)	(2.8)	(0.1)
Male	**4,453,700**	(87.8)	(8.9)	(3.2)	**5,236,551**	(1.6)	(95.6)	(2.7)	(0.1)

**Abbreviations:** AI/AN = American Indian/Alaska Native; NHPI = Native Hawaiian or other Pacific Islander.

* Vaccines administered during December 14, 2020–February 14, 2021, and reported to CDC by February 20, 2021.

^†^ Among persons who received their first dose on or before January 19 for Pfizer-BioNTech (>25 days between the first dose and February 14) or January 12 for Moderna (>32 days between the first dose and February 14).

^§^ 26–42 days (Pfizer-BioNTech) or 33–42 days (Moderna) after first dose; no second dose received.

^¶^ >42 days after first dose; no second dose received.

** Received second dose <17 days (Pfizer-BioNTech) or <24 days (Moderna) after first dose.

^††^ Received second dose 17–25 days (Pfizer-BioNTech) or 24–32 days (Moderna) after first dose.

^§§^ Received second dose 26–42 days (Pfizer-BioNTech) or 33–42 days (Moderna) after first dose.

^¶¶^ Received second dose >42 days after first dose.

*** Percentages were calculated among persons with available demographic characteristics.

Race/ethnicity was reported for 6,764,604 (54.1%) persons who had sufficient time to receive the second dose. Among persons for whom information on race/ethnicity was reported, demographic differences in completion status were also observed (Table 2) (Supplementary Figure 3, https://stacks.cdc.gov/view/cdc/103854). The lowest series completion rate (83.7%) and the highest prevalence of missing the second dose (5.1%) was among AI/AN persons. Series completion rates among non-Hispanic persons of multiple/other races (86.1%) and Hispanic persons (87.0%) were lower than the rates among non-Hispanic Native Hawaiian or other Pacific Islander (90.3%) and non-Hispanic White (90.3%) persons. Age was reported for >99% of vaccine recipients. Series completion was lowest among adults aged ≥65 years (87.2%); however, adults in this age group also had the lowest percentage of missed second doses (2.3%). Among persons aged 16–44 years, 4.0% missed the second dose. Differences in series completion and missed second doses by sex were minimal.

Among 14,205,768 persons who received a second COVID-19 vaccine dose, 95.6% received the second dose within the recommended interval, 1.5% received the dose early, 2.8% received it after the recommended interval but within the allowable interval, and 0.1% received the dose late. Differences in receipt of the second dose within the recommended interval were observed across jurisdictions (median = 96.4%; range = 79.0%–99.9%), with >10% of vaccine recipients receiving the second dose outside the recommended interval in four jurisdictions. A median of 1.0% of persons across jurisdictions received the second dose early (range = 0.1%–8.7%); >2% of persons received the second dose early in 10 of the 58 jurisdictions. Late receipt of the second dose ranged from 0.0% (14 jurisdictions) to 0.8% (median = 0.1%); approximately 0.4%–0.8% of vaccine recipients received the second dose late in three jurisdictions. Differences in receipt of the second dose within the recommended interval by demographic characteristics were minimal.

## Discussion

Two doses of the Pfizer-BioNTech and Moderna COVID-19 vaccines are required for optimal vaccine effectiveness (*1*). During the first 2 months of the U.S. COVID-19 vaccination program, among persons who received a first dose and had sufficient time to receive the second dose, 88.0% had completed the series and 3.4% had missed the second dose. Among all persons who received a second dose, the majority (95.6%) had done so within the recommended interval. These data are reassuring; however, the groups prioritized to receive vaccine during this period were more likely to have been vaccinated at their work site or residence, including health care workers (*2*) and long-term care facility residents*** (*3*), which might have facilitated adherence to the recommended schedule. As priority groups broaden, adherence to the recommended dosing interval might decrease. Although the second dose should be administered as close to the recommended interval as possible, it may be administered up to 42 days after the first dose when a delay is unavoidable.^†††^ If the second dose is administered beyond the allowable interval, the series does not need to be restarted.^§§§^ Providers should not administer second doses before the recommended interval or hold or save doses for patients who have not returned >42 days after their first dose. Providers should regularly assess missed second doses and repurpose those doses as first doses for eligible persons to initiate the vaccination series.

This interim analysis identified differences in completion status among jurisdictions and some demographic groups, findings that can be used to inform and enhance technical assistance for COVID-19 vaccination. Series completion was lowest among older adults, a finding that is similar to results from an initial nationwide examination of coverage (*4*). However, this group was also the least likely to miss the second dose; a large percentage remained within the allowable interval for the second dose. Among racial and ethnic groups, series completion was lowest among AI/AN persons, who also had the highest prevalence of missed second doses. To improve accessibility to and acceptance of second doses and maximize timely series completion, public health officials should work to better understand whether missed doses or delays are caused by challenges to vaccine access (e.g., supply, clinic availability, or community disadvantages) or because of other challenges related to vaccine confidence or acceptance.

The findings in this report are subject to at least four limitations. First, second-dose status was unknown for 7.9% of first-dose recipients (i.e., persons with an unknown manufacturer for the first dose and persons who lived in one state with limited data reporting). Second, persons might have been counted twice if they received doses from two different reporting entities or if their first and second doses were not linked because they were assigned a different recipient ID at their second-dose administration, possibly resulting in an underestimate of series completion. This jurisdiction mismatch could have contributed to the series completion and missed dose rates for AI/AN persons because tribal nations can border multiple jurisdictions and also because they might have received their vaccine through a separate allocation to the Indian Health Service. Conversely, if the same recipient ID was assigned to two or more persons in the same reporting entity, doses administered to two separate persons might have been coded as a first and second dose, possibly resulting in an overestimate of series completion. Third, several winter weather events led to canceled vaccination clinics and distribution challenges, which might have played a role in certain differences among jurisdictions. Finally, race/ethnicity data were missing for 45.9% of persons who had sufficient time to receive the second dose, limiting the ability to interpret differences in vaccination completion and timing of receipt of the second dose by race/ethnicity.

Nearly 9 in 10 persons with sufficient time to receive their second COVID-19 vaccine dose completed the series and did so within the recommended interval. Missed doses and second doses administered outside the recommended interval were infrequent but varied by jurisdiction and demographic groups. Public health officials and providers should work to better understand the reasons for lack of completion of the COVID-19 vaccination series and early and delayed intervals. To ensure completeness and equity in series completion, CDC provides technical assistance^¶¶¶^ to improve strategies for completion within the recommended time intervals. Jurisdictions can work with providers to prioritize second doses to ensure vaccination series completion, reschedule persons whose vaccination appointments were canceled, repurpose missed second doses, and promote the importance of receiving a second dose for achieving maximum vaccine effectiveness. Providers can focus on support strategies such as scheduling follow-up visits during initial scheduling or first-dose administration and sending reminder notices before and after the recommended second-dose interval. Continued monitoring of series completion status across jurisdictions and by demographic characteristics is important to ensure equity in vaccine administration and vaccination coverage, especially as vaccination efforts expand to additional population groups.

**Summary**

**What is already known about this topic?**

During December 2020, two 2-dose COVID-19 vaccines received Emergency Use Authorization from the Food and Drug Administration.

**What is added by this report?**

Among persons who received a first dose and for whom sufficient time had elapsed to receive the second dose, 88.0% had completed the series; 8.6% had not received the second dose but were still within the allowable interval to receive it. Among all 2-dose recipients, 95.6% received the second dose within the recommended interval. Differences in missed doses or second doses administered outside the recommended interval were identified among jurisdictions and demographic groups.

**What are the implications for public health practice?**

Identifying and addressing possible barriers to completing the COVID-19 vaccination series can help ensure equitable coverage across communities and optimal health benefits for recipients.

## Footnotes

†
https://www.fda.gov/emergency-preparedness-and-response/coronavirus-disease-2019-covid-19/moderna-covid-19-vaccine

§ Data on the efficacy of mRNA COVID-19 vaccines beyond this window are limited. If the second dose is administered beyond these intervals, the current recommendation is that the series does not need to be restarted. https://www.cdc.gov/vaccines/covid-19/info-by-product/clinical-considerations.html

¶
https://www.cdc.gov/vaccines/covid-19/vaccination-provider-support.html

** Each jurisdiction that submits COVID-19 vaccine administration data to CDC is assigned a three-digit reporting entity code that references the entity that submitted the vaccination record to CDC.

†† Texas did not submit individual-dose vaccination data; therefore, the 3,096,281 persons who received ≥1 dose in Texas were excluded. Five federal agencies (Federal Bureau of Prisons, U.S. Department of Defense, U.S. Department of State, Indian Health Services, and Veterans Health Administration) and 21 pharmacy outlets received a direct allocation of vaccine and are included in this analysis within state totals.

§§ 45 C.F.R. part 46, 21 C.F.R. part 56; 42 U.S.C. Sect. 241(d); 5 U.S.C. Sect. 552a; 44 U.S.C. Sect. 3501 et seq.

¶¶ The cut-off values were selected to identify the approximately 10%–15% of jurisdictions with outlier values.

***
https://www.cdc.gov/coronavirus/2019-ncov/vaccines/recommendations.html

†††
https://www.cdc.gov/vaccines/covid-19/info-by-product/clinical-considerations.html

§§§
https://www.cdc.gov/vaccines/covid-19/vaccine-inventory-management.html

¶¶¶
https://www.cdc.gov/vaccines/covid-19/toolkits/index.html
